# Physical Activity as a Modifiable Risk Factor for Gastroesophageal Reflux Disease in Saudi Arabia: A Systematic Review and Meta-Analysis

**DOI:** 10.7759/cureus.85271

**Published:** 2025-06-03

**Authors:** Abdulrahman M Albeshry, Saud Feaz Althobaiti, Salem Almerri, Najeeba Sayed, Sara A Alghamdi, Ibtihal N Albalwai, Sarah Yahya S Mones, Amani Yasr Alsharari, Shahad Amjd khodri, Suzan Mohammed Bukhari, Shouq Rashed Aljohani, Lama Saeed Alghamdi, Khaled Hesham Albahkali, Ruba Ibrahim Alghannami, Hatim Zohair Khawandanh

**Affiliations:** 1 Family and Community Medicine, Faculty of Medicine, University of Jeddah, Jeddah, SAU; 2 Internal Medicine, King Abdulaziz Medical City, Ministry of National Guard, Riyadh, SAU; 3 General Medicine, Farwaniya Hospital, Kuwait, KWT; 4 General Medicine, King Fahad Specialist Hospital, Manama, BHR; 5 Medicine, King Saud Bin Abdulaziz University for Health Sciences, Jeddah, SAU; 6 Medicine, Al Rayan National College of Medicine, Madinah, SAU; 7 Medicine, King Khalid University, Abha, SAU; 8 Medicine, College of Medicine, Jouf University, Sakaka, SAU; 9 Medicine, College of Medicine, University of Jeddah, Jeddah, SAU

**Keywords:** gastroesophageal reflux disease, gerd, physical activity, risk factor, risk factors, saudi arabia

## Abstract

Gastroesophageal reflux disease (GERD) is one of the most common disorders, significantly impacting patients' quality of life. Research suggests that physical activity (PA) can substantially reduce the occurrence of GERD. This systematic review and meta-analysis aim to investigate the association between PA and GERD in Saudi Arabia. The databases searched included Google Scholar, PubMed, Web of Science, Cochrane Library, Medline, Embase, NCBI, Scopus, and regional sources such as the Saudi Digital Library and Index Medicus for the Eastern Mediterranean Region. Cross-sectional and quasi-experimental studies conducted in Saudi Arabia between 2015 and 2024 were included to explore the relationship between PA and GERD. The quality of the included studies was assessed using the Newcastle-Ottawa Scale. A meta-analysis was conducted using MetaXL version 5.3 (EpiGear International Pty Ltd., Queensland, Australia), employing a random-effects model to estimate the overall prevalence of GERD. The OR was used to assess the relationship between PA and GERD. Heterogeneity was evaluated using the Higgins I² statistic and tested with the Cochran Q test. A total of 250 studies were included, comprising 6,183 participants. The pooled prevalence of GERD was estimated at 35% (95% CI: 24-47%). Individuals with lower levels of physical activity were 22% more likely to develop GERD compared to those with higher activity levels (pooled OR = 1.22, 95% CI: 1.05-1.42). The analysis revealed a high level of heterogeneity among the studies (I² = 99%, p < 0.001). Five studies were rated as high quality, while two were of medium quality. In conclusion, low physical activity increases the risk of GERD in Saudi Arabia. PA appears to be an effective primary prevention strategy to reduce the incidence of GERD. Further research is needed to confirm these findings and clarify the underlying mechanisms.

## Introduction and background

It was found that nearly 10% to 15% of the general population in the world suffers from gastroesophageal reflux disease (GERD) [1,2]. Among all cases, the highest prevalence reported in Saudi Arabia is approximately 45.4% among older individuals attending primary care in Riyadh [3]. The symptoms of GERD are a burning sensation, acidity, regurgitation, and reflux of the gastric contents [4]. Unless treated on time, GERD can alter the lifestyle of an individual and predispose the affected person to develop Barrett’s esophagus or esophageal adenocarcinoma. Hence, early disease diagnosis is crucial for implementing effective prevention and management procedures [5].

Saudi Arabia has undergone socioeconomic developmental changes over the past few decades, including urbanization, shifts in food habits, and, more recently, sedentary lifestyles, all of which predispose individuals to GERD [6]. This new lifestyle, mainly defined by unhealthy consumption of high-fat and sugar diets and reduced physical activity (PA), has been correlated with the rise in obesity, which is a risk factor for GERD. In this context, the role of obesity in GERD is pertinent, as obesity leads to increased intra-abdominal pressure and changes in the lower esophageal sphincter [7]. Such alterations in societal and lifestyle status assert that GERD poses a rising concern to public health in Saudi Arabia and, more specifically, call for research aimed at understanding the analysis and modifiable risk factors [8].

PA has been identified as a protective factor against GERD. Low-impact exercise is said to enhance stomach motility, promote gastrointestinal motility, and decrease stomach pressure; therefore, it may aid in alleviating GERD symptoms [9]. There is a two-way interaction between PA and GERD, whereby the two are moderated by factors such as intensity and kind of exercise. While PA may reduce the risk of developing GERD, high-intensity exercises, including those that cause intense abdominal muscle contractions, have been said to worsen the symptoms in some patients. The complex relationship is fundamental to creating effective recommendations for lifestyle changes related to GERD [10].

It has been noted that PA has benefits in the early prevention and treatment of chronic diseases [11,12]. It is not clear how PA is related to GERD. As highlighted by Amiri and Zemková, PA has been discovered to enhance the diaphragm activity and, thus, the anti-reflux barrier [13]. The current socioeconomic changes have led to significant alterations in the quality of life, including shifts in patterns of PA and nutrition. Furthermore, routine PA may help prevent GERD in individuals who are either obese or have a normal body mass index (BMI) [14].

The current socioeconomic changes are responsible for the deteriorated quality of life, primarily through factors such as changes in PA patterns and diet. One of the most common risk factors of GERD is the lack of a healthy lifestyle and harmful habits such as smoking, which leads to complications such as obesity [15]. The lifestyle of the individuals was severely affected by the COVID-19 pandemic, potentially leading to the chances of GERD [16,17].

Though there is a higher prevalence rate of GERD in Saudi Arabia, and PA has been found to play a significant part in the management, there are not enough studies that support the positive correlation between GERD and PA. The current systematic review and meta-analysis aim to fill the gap by assessing the risk factors of GERD and the frequency of occurrence among physically active individuals and the sedentary population in Saudi Arabia. Moreover, this study examines the impact of PA on symptoms. The current review provides a detailed understanding of the relationship between PA and GERD and offers valuable insights for clinical professionals. Therefore, the study helps take valuable steps to prevent and treat individuals diagnosed with GERD.

## Review

Methods and materials

Search Strategy

Google Scholar, PubMed, Web of Science, Cochrane Library, Medline, Embase, NCBI, and Scopus were the databases used to pull data from 2015 to 2024. The following keywords were used for the search: GERD, physical activity, and exercise. It was discovered that all the developed question prompts were accepted by the Medical Subject Headings (MeSH) Library. The appropriate terminologies were connected using the AND and OR Boolean operators to produce a widened search method. Other regional databases, including the Saudi Digital Library and Index Medicus for the Eastern Mediterranean Region, were also utilized.

Eligibility Criteria

For the inclusion criteria, cross-sectional studies investigating the association between PA and GERD in Saudi Arabia were included. Relevant studies provide comprehensive information on the prevalence of GERD, diagnostic strategies, and PA. Studies conducted in Saudi Arabia in either English or Arabic were included.

For the exclusion criteria, non-observational studies, including clinical trials, reviews, or case reports; studies that emphasized certain subpopulations; or studies deemed irrelevant were excluded. Studies executed in places other than Saudi Arabia and published in languages other than English or Arabic were also excluded.

Study Selection

A systematic strategy was applied to comprehensively recognize and evaluate the included studies. The selected studies were transferred to EndNote (Clarivate, London) to eliminate any similar studies. The two eligible reviewers evaluated and screened the titles and abstracts and reviewed the entire study text. Any reviewer conflicts were resolved through discussion or consultation with a third reviewer when necessary.

Data Extraction

Information was gathered from the selected studies using a developed data extraction form. The extracted data sources comprised the author, the year of publication, design, population, sample, prevalence, definition of GERD, diagnosis, and PA of GERD patients.

Quality Assessment

While analyzing the quality of the included studies, cross-sectional studies were evaluated based on the Newcastle-Ottawa Scale (NOS) [18]. This scale evaluates studies based on three domains; however, comparability and exposure/outcome tend to arise where selection bias occurs. Considering the suggested approach, the range of scores was determined as follows: 7 to 9 points indicate high-quality studies, 5 to 6 points indicate medium-quality studies, and scores less than 5 indicate low-quality studies. Two authors rated each study, and the issue was discussed in cases of between-rater disagreement to reach a consensus.

The NOS for cross-sectional studies assessed the study's quality. This scale evaluates studies based on selectivity, comparability, and exposure/outcome. The range of scores was determined as follows: 7 to 9 points indicate high-quality studies, 5 to 6 points indicate medium-quality studies, and scores less than 5 indicate low-quality studies. The two reviewers assessed each study, and any conflicts or disagreements were resolved through conversation.

Statistical Analysis

MetaXL version 5.3 (EpiGear International Pty Ltd., Queensland, Australia), coupled with Python, was used in the statistical analysis of the data. This approach enabled substantial statistical analysis of the collected data and provided accurate graphic visualization of the achieved outcomes. In the present meta-analysis, a random-effects model using the inverse variance method was employed to estimate the overall prevalence of GERD and to assess the relationship between PA and GERD. MetaXL was used to combine the prevalence of GERD among participants identified in the included studies, and the results were presented in a forest plot. To evaluate the association between PA and GERD, the odds ratio (OR) with its 95% confidence interval (CI) for each study was estimated, and these ORs were combined using a random-effects model. Python was used to create forest plots of the results, formatting them to enhance the display of the results on the software.

Interstudy variability was also assessed using the Higgins I² statistic in MetaXL, where values above 50% indicated significant interstudy variability. Therefore, Cochran's Q test was also used to check the heterogeneity of the results. Publication bias was analyzed with the help of funnel plots and Egger's test. There are no restrictions regarding the type of statistical tests for this study. All significance tests were two-tailed, and the level of statistical significance was set at p < 0.05.

Results

Article Screening and Selection

**Figure 1:**
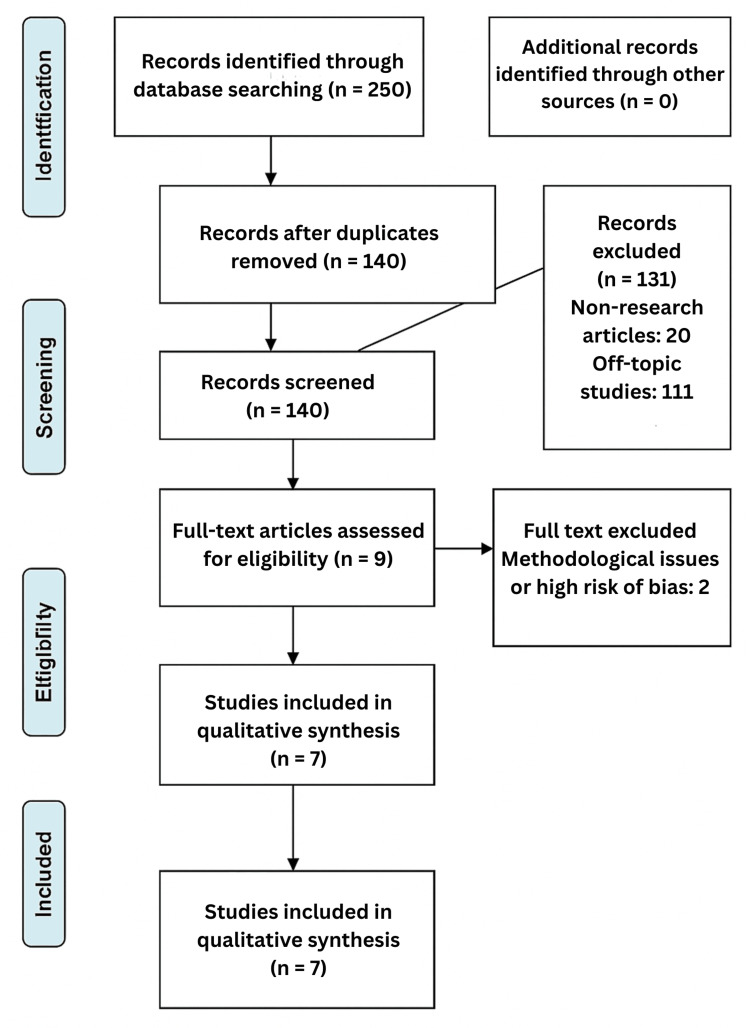
Flow diagram of the article screening and selection process

A total of 250 articles were initially identified through database searching (Figure 1). After the removal of duplicates, 140 articles remained for screening. One hundred thirty-one were excluded, including 20 non-research articles and 111 off-topic studies. Nine studies were subject to a full-text evaluation, with two excluded due to methodological concerns or a high risk of bias. Seven studies met the inclusion criteria and were included in the qualitative synthesis.

Characteristics of the Included Studies

The seven studies included in this review were all cross-sectional, examining the relationship between PA and GERD in diverse populations across Saudi Arabia (Table 1).

**Table 1:** Characteristics of the studies included in this review GERD: gastroesophageal reflux disease, PA: physical activity

Study	Study design	Population	Sample size	GERD prevalence	GERD diagnosis method	PA among GERD patients	PA definition
Alkhathami et al., 2017 [19]	Cross-sectional study	General Saudi population	2043	28.7% (587 participants)	GERDQ questionnaire (Score ≥8)	248 (42.3% of GERD patients)	Exceeding 30 minutes per week
Alrashed et al., 2019 [20]	Cross-sectional study	Students at Shaqra University	400	23.75% (95 participants)	GERDQ questionnaire (Score ≥8)	31 (32.6% of GERD patients)	Exceeding 30 minutes per week
Al-Towairqi et al., 2020 [21]	Cross-sectional study	Female medical students at Taif University	256	29.3% (75 participants)	GERDQ questionnaire (Score ≥8)	12 (16% of GERD patients)	More than 5 times per week
Al Ghadeer et al., 2021 [22]	Cross-sectional study	General population from the Eastern Province of Saudi Arabia	1517	20.57% (312 participants)	GERDQ questionnaire (Score ≥8)	131 (42% of GERD patients)	Exceeding 30 minutes per week
Kuddus et al., 2021 [23]	Cross-sectional study	The general population of Hail City	704	57.95% (408 participants)	GERDQ questionnaire (Score ≥8)	217 (53.2% of GERD patients)	More than once per week
Alsaleem et al., 2021 [24]	Cross-sectional study	Adults in Abha attending primary healthcare centers	320	67.81% (217 participants)	GERDQ questionnaire (Score ≥8)	194 (89.4% of GERD patients)	Exceeding 30 minutes per week
Otayf et al., 2022 [25]	Cross-sectional study	Jazan University students in Jazan Province	953	23.08% (220 participants)	GERDQ questionnaire (Score ≥8)	129 (58.6% of GERD patients)	Exceeding 30 minutes per week

While these studies considered various risk factors for GERD, the primary focus was on evaluating how different levels of PA influenced the prevalence and severity of the disease. These studies analyzed 6,193 participants, with sample sizes ranging from 256 to 2,043 individuals. The prevalence of GERD among these populations showed considerable variation, ranging from 20.57% to 67.81%, reflecting the differing demographic and lifestyle factors of each study. The GERDQ questionnaire, with a score of 8 or higher indicating a diagnosis of GERD, was adopted as the standard diagnostic tool used across all studies, ensuring consistency in the identification of GERD. PA levels among GERD patients were comprehensively reported, with participation rates spanning from as low as 16% to as high as 89.4%. However, the criteria for defining PA varied, with some studies considering "more than 30 minutes per week" sufficient. In contrast, others set a higher threshold, such as "more than five times per week."

GERD Prevalence Among Participants

**Figure 2:**
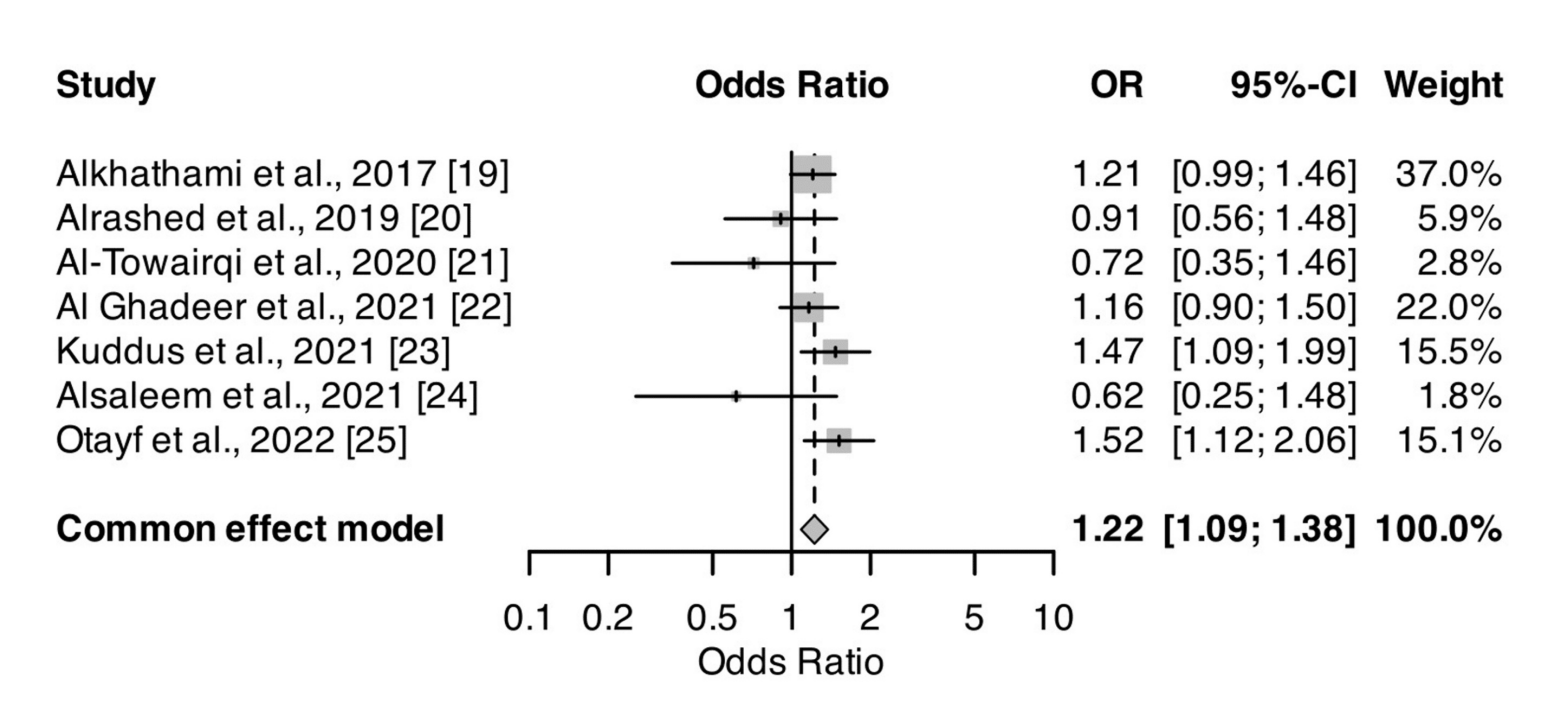
Forest plot showing the prevalence of GERD among participants in the included studies Blue square boxes represent the rate. GERD: gastroesophageal reflux disease, OR: odds ratio, CI: confidence interval

Figure 2 provides a forest plot depicting the prevalence of GERD across the included studies. The blue square boxes illustrate the prevalence rates, indicating considerable variability, with estimates ranging from 21% to 68% across various populations. The pooled analysis yielded a GERD prevalence of 35% (95% CI: 24-47%), suggesting that over one-third of the study participants were affected by GERD. The high degree of heterogeneity (I² = 99%) highlights substantial differences between studies, likely due to variations in sample populations, diagnostic criteria, and geographic or lifestyle factors.

Odds Ratios for Low Physical Activity in GERD Patients

**Figure 3:**
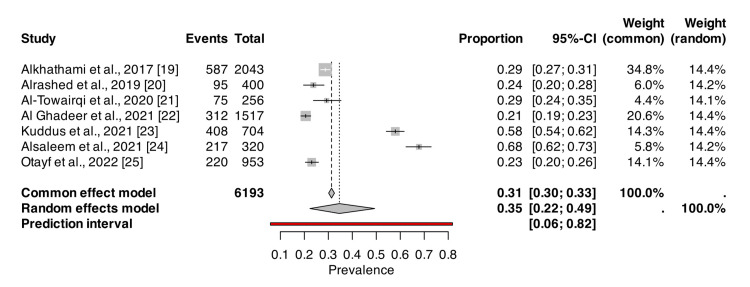
Forest plot of ORs for PA in patients with GERD from the included studies ORs: odds ratios, PA: physical activity, GERD: gastroesophageal reflux disease, CI: confidence interval

Figure 3 presents a forest plot of ORs for low PA among GERD patients across the included studies. The pooled OR of 1.22 indicates that individuals with lower PA levels have a 22% higher likelihood of developing GERD than those with higher activity levels. The CIs vary across studies, with some, like Kuddus et al., showing a stronger association (OR = 1.47). In contrast, others, like Al-Towairqi et al., suggest a weaker or inverse relationship (OR = 0.72).

Quality Assessment of the Included Studies

The quality assessment of the included studies, as summarized in Table 2, indicates that five of the seven studies were classified as high quality, with total scores ranging from 7 to 9 out of a maximum of 9 points. These studies demonstrated strong selection processes, effective control of confounders, and robust outcome assessments. However, two studies (Al Ghadeer et al. and Alsaleem et al.) were rated as medium quality, with scores of 6 and 5, respectively. These studies had limitations in their selection criteria or comparability, slightly reducing their overall quality ratings.

**Table 2:** Quality assessment of the included studies

Study (author, year)	Selection (max 4 points)	Comparability (max 2 points)	Exposure/outcome (max 3 points)	Total score (max 9 points)	Quality tier
Alkhathami et al., 2017 [19]	3	2	2	7	High
Alrashed et al., 2019 [20]	4	2	3	9	High
Al-Towairqi et al., 2020 [21]	3	2	2	7	High
Al Ghadeer et al., 2021 [22]	3	1	2	6	Medium
Kuddus et al., 2021 [23]	3	2	3	8	High
Alsaleem et al., 2021 [24]	2	1	2	5	Medium
Otayf et al., 2022 [25]	4	2	3	9	High

Discussion

The present systematic review and meta-analysis aim to investigate the association between PA and GERD in Saudi Arabians. GERD is the most frequently occurring gastrointestinal illness, characterized by significant morbidity. Therefore, it is essential to recognize the risk factors that are vital for managing the disease [26]. The PA is widely recognized as the most cost-effective intervention for reducing risk factors associated with various chronic illnesses. According to guidelines, at least two hours and 30 minutes of moderate exercise or one hour and 15 minutes of vigorous aerobic exercise are required weekly to achieve considerable health benefits [27]. PA assists in treating heart diseases, diabetes, and specific gastrointestinal disorders, including irritable bowel syndrome-related symptoms, stomachaches, and bloating [28].

However, while PA has been advocated to help prevent many gastrointestinal diseases, there is a relative lack of certain specific guidelines outlining the role of PA in the treatment of GERD [29]. This cross-sectional design identified cases of GERD, particularly in the region of Saudi Arabia, and the current systematic review and meta-analysis have addressed this gap. The cases of GERD are particularly high in the region of Saudi Arabia, and the current systematic review and meta-analysis have taken into account this gap. The study results help to better understand the link between PA and GERD within a certain group of people [30].

According to the past literature, there is a greater prevalence of gastrointestinal diseases such as GERD in Saudi Arabia. As per studies conducted by Alkhathami et al. and Al Ghadeer et al., the prevalence of GERD is 28.7% and 20.57%, respectively [19,22]. On the contrary, Alsaleem et al. reported a greater prevalence rate of 67.81% [24]. In addition, studies performed in the United States, including research by Coleman et al., illustrated a prevalence rate of GERD near 20-25% and reported that regional factors, including dietary habits, strongly influence the prevalence of GERD; in our study population, the typical diet was high in fat and sugar, which may have contributed to the observed prevalence [31].

The relationship between PA and GERD varied for each category. The current study calculated the pooled OR to be 1.22, indicating that participants with lower PA levels are 22% more likely to develop GERD. This observation was further supported by El-Serag et al., who argued that increased PA levels helped reduce GERD by 15-20% [1]. Specifically, Kuddus et al. estimated the same value in our analysis, 1.47, which highlights that sedentary behavior increases the chance of GERD [23].

On the other hand, some studies have shown a weaker or even inverse association between PA and GERD. For example, Djärv et al. reported that intermediate levels of PA were associated with a reduced occurrence of GERD among obese individuals (adjusted OR: 0.41; 95% CI: 0.22-0.77), while no significant effect was observed in non-obese individuals [27-36]. Similarly, Kuddus et al. found that higher daily step counts were linked to a lower risk of incident GERD (HR per 1,000 steps = 0.78; 95% CI: 0.66-0.92), with the protective effect consistent across different BMI strata. These findings highlight the variability in PA-GERD associations, which may be influenced by confounding factors such as BMI and dietary patterns [23]. The same disparities are observed using PA's interaction with GERD across BMI status, dietary patterns, and health status in other countries. For example, the study by Wu et al. established that PA was highly protective, especially in participants with a high BMI, as opposed to those with a normal BMI, for whom the survival benefit was minimal. This implies that the relationship between PA and GERD could be rather complex and depends on weight status [32].

A key limitation of prior research is the heterogeneity in the concept of PA across studies. In the present review, the criteria vary from "more than 30 minutes per week," as illustrated by Alkhathami et al., to "more than five times per week," as reported by Al-Towairqi et al. [19,21]. The PA and other factors, such as BMI and diet, have been observed to be advantageous for reducing the risk of developing GERD [14]. Similar to the findings of the current research and those of Alsaleem et al., PA may be even more advantageous to overweight or obese people. This finding aligns with the results of Mosa et al., who reported that weight loss combined with exercise results in the lowest GERD symptoms [33]. On the other hand, in normal weight groups, including Al-Towairqi's study, the advantage was not noticeable [21].

The PA is a crucial factor in the reduction of GERD symptoms; however, the individuals suffering from GERD involved in the PA differ considerably [37]. As reported by Al-Towairqi et al., 16% of the GERD patients are involved in PA. Alsaleem et al. reported that 89.4% of individuals engaged in PA [21,24]. Although there are variations, PA is beneficial in reducing the risk factors associated with GERD. This result aligns with a previous study, which suggests that a moderate level of PA can aid in managing GERD by enhancing esophageal function, reducing intra-abdominal stress, and improving gastric emptying [38]. Despite the positive aspects of PA, a study suggests that excessive muscle pulling and activity can lead to GERD, primarily due to increased intra-abdominal stress and alterations in the lower esophageal sphincter during intense exercises [39].

The protective effect of PA against GERD has enormous public health considerations, especially in Saudi Arabia, where people are leading sedentary lives and obesity [40]. In this regard, the recommendation of PA as a preventive strategy for developing GERD may have significant implications, as it offers a preventive approach for preventing the development of other diseases and alleviating the suffering of patients with GERD [41]. This study suggests that improved health education and exercise promotion, aligned with Saudi Arabian cultural expectations, may help reduce the burden of GERD. Adopting PA guidelines in the primary healthcare management of GERD may also lead to increased practice of the recommendations [42].

Further longitudinal research is needed to establish a direct correlation between PA and GERD. This article highlights several recommendations: using devices for PA, specifically accelerometers, can enhance measurement quality, and new diagnostic tools, such as endoscopy, should also be considered.

The generality of the study populations is another major concern. The majority of existing works have addressed specific categories of the population, such as university students or attendees at healthcare centers, and thus cannot accurately reflect the situation in Saudi Arabia as a whole. For instance, Alrashed et al. sampled students from Shaqra University, whereas Al-Towairqi et al. targeted female medical students at Taif University. These groups are potentially likely to differ from the general population in terms of age, lifestyle, and health-related practices, which may also restrict the generalizability of the results [20,21]. Due to the limitations of the current study, future researchers should strive to recruit participants from all age ranges and genders, with a range of income levels, and from different states within the United States.

A comparison between Saudi studies and other literature reveals the following similarities and differences. In the systematic review by Zhang et al., the authors found that moderate PA was inversely related to the prevalence of GERD, thereby confirming the idea that lifestyle interventions can be effective tools in preventing and treating the disease [14]. Still, according to the abovementioned high prevalence rates in Saudi Arabia, local factors like diet, obesity, and the endowment of PA can magnify the risk of GERD in this population, which was seen in Alsaleem et al. [24].

While contributing valuable data specific to the Saudi Arabian context, our review shares some limitations with global studies. These include reliance on self-reported PA and GERD measures, which may introduce recall bias, and the heterogeneity in PA definitions across studies, complicating the interpretation of results. Furthermore, the cross-sectional design of most included studies limits the ability to establish causal relationships between PA and GERD. Future research should aim to overcome these limitations by employing longitudinal designs and standardized PA measurements to provide more definitive evidence on the role of PA in GERD prevention and management.

## Conclusions

This study shows that there is a clear relationship between a low level of PA and high GERD prevalence in the Saudi Arabian population. These findings suggest that the benefits of PA in preventing and treating GERD are maintained even when PA is moderate in intensity. Given the high prevalence of GERD in Saudi Arabia, encouragement of PA could be viewed as a fundamental public health intervention. This calls for more research to identify how PA works in relation to GERD, as well as to come up with different recommendations based on age and sex. Moreover, it is suggested that further research should focus on developing evidence-based practice recommendations for PA in GERD prevention, with the assurance of applicability to various populations.
